# Fellow-Workers and Their Doings

**Published:** 1914-06-06

**Authors:** 


### ROUND THE WORLD.

### Fellow-Workers and their Doings.

[The Editor Invites Early Information and Contributions Marked "Fellow-Workers."]

Sir Felix Semon recently enjoyed the somewhat rare distinction of presiding at a London University lecture named after himself and founded in commemoration of his services to laryngology. Medical men owe it largely to the work of Sir Felix Semon that the study of diseases of the throat has been placed on a scientific basis, and it may be recalled that the foundation of the Laryngological Society in London was also largely due to his energies. Sir Felix took part in the Franco-German war as a volunteer and was present at several important engagements. An M.D. and F.R.C.P. Lond., he became physician extraordinary to the late King Edward VII. in 1897, and was created a K.C.V.O. in 1905.

As Principal and Vice-Chancellor of Glasgow University, Sir Donald MacAlister, who presided last week over the bi-annual session of the General Medical Council, holds an influential position in one of our great centres of medical education, whilst his previous experiences in various important posts have brought him into touch with practically every phase and problem of medical practice. In his early years, Sir Donald MacAlister, who is an old "Bart.'s" man, was a distinguished mathematician, being Senior Wrangler at Cambridge in 1877; he is also an M.D. Cantab., and F.R.C.P. Lond., besides possessing a host of honorary degrees.

Dr. Wu Lien Teh, M.D. Cantab., who is urging the reform of medical education in China, is one of the most interesting personalities on the British Medical Register. As Gnoh Lean Tuck he is known to many friends in this country, having studied medicine at Cambridge, and afterwards at St. Mary's Hospital, where for a short while he was a popular resident. Quite early in his career Dr. Wu Lien Teh showed a grasp of Western scientific methods and a keenness for research that enabled him at times to outrival his European colleagues. Thus at Cambridge he gained an exhibition and scholarship at Emmanuel College, whilst at St. Mary's he carried off the Kerslake Scholarship in pathology and the Cheadle gold medal for clinical research. Three years ago Tuck was chief medical officer to the Manchurian Plague Organisation and president of the International Plague Conference. He is at present director and chief medical officer to the Northern Manchurian Plague Prevention Service, and a respected authority amongst his countrymen on all health subjects.

Everyone's sympathy goes to Mr. Maynard Heath, who has just experienced bereavement through a terrible accident. Mr. Maynard Heath is a University College Hospital man, who is assistant surgeon to the Metropolitan Hospital and Evelina Hospital for Sick Children and surgeon to the Gordon Hospital; he is an F.R.C.S. Eng. and M.S. Lond., winning the gold medal at the examination for the last-mentioned diploma in 1907.

Mr. Robert Jones, who delivered the annual oration before the Medical Society of London the week before last, chose a subject of great importance when he decided to deal with the "Surgical Aspect of Poliomyelitis." Mr. Jones is a well-known surgeon in Liverpool, where he is a member of the staffs of the Royal Southern Hospital and the Royal Country Hospital for Children, as well as lecturer on orthopædic surgery at the University. Qualifying from Liverpool and Dublin as L.R.C.P. Edin. in 1884, he became an F.R.C.S. Edin. five years later, and was subsequently awarded the honorary distinctions of Ch.M. Liverpool and F.R.C.S.I. For many years Mr. Robert Jones has given special attention to the treatment of infantile paralysis.

An interesting subject was dealt with by Dr. T. Wilson Parry at the recent meeting of the North London Medical and Chirurgical Society, when he gave the results of his historical researches into the ways in which pre-historic man endeavoured to help himself when attacked by sickness or accident. In a review of the earliest days of human existence Dr. Parry reminded his audience that primitive man probably did nothing more than follow the ordinary practice of animals and lick his wounds, pointing out very fully that such licking is really a process of crude fomentation and massage.

What appears to be a useful method of assisting certain cases of tuberculosis is being worked out by Dr. J. H. Whelan. Some two years ago he drew attention to satisfactory results obtained by a combined strychnine-tuberculin treatment, in which the tuberculin was given once a week and the strychnine injected intramuscularly every day. Dr. Whelan now reports (*British Medical Journal*, May 16) further results obtained by a similar method, in which, however, larger doses of strychnine have been followed by increased benefits; he points out that the daily intramuscular injections of strychnine are not as disadvantageous as might be thought, and advises a dose of 18 to 25 minims of a 1 in 400 strychnine solution. Dr. Whelan was formerly in the Royal Navy Medical Service, retiring with the rank of Fleet Surgeon.

The Royal Hospital for Diseases of the Chest, in the City Road, owes a great deal to Dr. Barty King, the dean of the new Medical School there, whose efforts have been largely responsible for the inflow of funds that have rendered possible the establishment of the new laboratories and improvements at that institution. Dr. Barty King gained the gold medal at the M.D. examination at Edinburgh University in 1902, and is a member of the Royal Colleges of Physicians both in London and Edinburgh. Having obtained experience of pulmonary tuberculosis in various resident appointments, he settled in practice in the West End, and, becoming attached to the Royal Hospital for Diseases of the Chest and to the St. Pancras Dispensary as physician for chest diseases, has had excellent opportunities of developing his specialty.

